# Social-Economic Status and Cognitive Performance among Chinese Aged 50 Years and Older

**DOI:** 10.1371/journal.pone.0166986

**Published:** 2016-11-18

**Authors:** Fan Wu, Yanfei Guo, Yang Zheng, Wenjun Ma, Paul Kowal, Somnath Chatterji, Ling Wang

**Affiliations:** 1 School of Public Health, Fudan University, Shanghai, China; 2 Shanghai Institute of Preventive Medicine, Shanghai, China; 3 Shanghai Municipal Center for Disease Control & Prevention, Shanghai, China; 4 World Health Organization, Department of Health and Information Systems, Geneva, Switzerland; 5 University of Newcastle Research Centre for Generational, Health and Ageing, Newcastle, Australia; 6 Guangdong Provincial Institute of Public Health, Guangdong Provincial Center for Disease Control and Prevention, Guangzhou, China; Soochow University Medical College, CHINA

## Abstract

**Background:**

Numerous population-based studies have suggested that socio-economic status (SES) is associated with cognitive performance, but few nationally representative epidemiological studies on cognitive performance with a large sample of older adults are available in China. And many studies explore the factors associated with cognitive performance, mainly focusing on individual level and more rarely on multiple levels that include the individual and community.

**Methods:**

This study uses SAGE-China Wave 1 data which consisted of 13,157 adults aged 50 years and older to explore socioeconomic inequalities in the cognitive performance from a multilevel perspective (individual and community levels). The overall cognition score was based on the seven separate components of the cognition tests, including the four verbal recall trials, the verbal fluency test, the forward digit span test and the backward digit span test. Factor analysis was applied to evaluate and generate a single overall score. A two-level hierarchical linear model was used to evaluate the association between SES at these two levels and the overall cognition score adjusted for age, sex and marital status.

**Results:**

At individual level, years of education was significantly associated with overall cognition score for both urban and rural dwellers. At the community level, a positive association was obtained between median household income and median years of education and overall cognition score among urban participants.

**Conclusion:**

A significant association between SES at both individual-level and community-level (only for urban area) and cognitive performance were found in this study of a national sample of 13,157 Chinese aged 50 years and older, even after adjusting for demographic characteristics. Identifying community-based SES variables that are associated with cognitive performance in the older population provides further evidence for the need to address community characteristics associated with deprivation.

## Background

Cognitive function refers to those mental processes that are crucial for the conduct of activities of daily living. Such mental processes include attention, memory, reasoning, coordination of movement and planning of tasks[1]. A number of components of cognitive function typically decline with ageing. Cognitive impairment, including dementia as one outcome of decline, may increase considerably with the rapidly growing population of older adults. It is a major health and social problem worldwide, including China, affecting loss of independence in daily activities and lowers the quality of life in older age[2, 3].

A number of population-based studies have suggested that socio-demographic characteristics is associated with cognitive performance, with increasing age, female sex and lower education consistently associated with lower cognitive performance [4, 5]. Cognitive decline is also associated with poor financial status [6, 7]. Socioeconomic inequalities have also been shown to contribute to discrepancies in cognition.

Compared with higher income countries, few nationally representative epidemiological studies on cognitive performance with a large sample of older adults are available in China. On the other hand, many studies explore the factors associated with cognitive performance, mainly focusing on individual level and more rarely on multiple levels that include the individual and community. One study among community-dwelling older adults in Singapore showed that neighborhood socioeconomic status is related to cognitive performance [7]. Living in a low-SES community is independently associated with cognitive impairment in an urban Asian society. Another study conducted in older US women supported the view that the socioeconomic status of a woman's neighborhood is associated with cognitive performance [8]. These studies point to additional factors, community effects, which should be drawn into any investigation of cognition.

The Study on global AGEing and adult health (SAGE) China Wave 1 is the baseline of a longitudinal population-based survey designed to provide high quality health and well-being data on the adult population and ageing process in China. To our knowledge, this was the first nationwide population-based older adults’ health and well-being survey in China. This study uses SAGE-China Wave 1 data to examine whether socioeconomic status is related to cognitive test performance from a multilevel perspective (individual and community levels). In addition, we assessed how much of the variation in cognitive test performance was explained by socioeconomic variables.

## Method

### Study population and survey design

The WHO Study on global AGEing and adult health (SAGE) aims to understand the Impact of aging on health and well-being in China and five other countries including Ghana, India, Mexico, Russia and South Africa. SAGE-China involves a nationally representative sample of adults aged 50 years and older, their spouses, and other persons aged 50 years and older in the household (n = 8,000). A probability sampling design and a five-stage cluster sampling strategy were used. First, eight provinces/municipality were selected from a total of 31 provinces/municipalities in China, according to its geographic area and social economic level. Second, one rural county and one urban district in each province/municipality were selected. In total, eight provinces and 16 strata (county/district) were selected for SAGE-China. The study sample covered a total of 64 principle sample units (PSU) (four urban and four rural township/community from each county/district), 128 secondary sample units (SSU) (two villages/EAs per township/community) and 256 tertiary sample units (TSU) (two residential blocks per villages/EAs). Wave 1 was successfully completed in 2010, and consists of 10,218 “50 years and older” households and 13,177 individual respondents aged 50 years and older. The details were specified in the appendix S1 Appendix.

### Ethics and consent

WHO’s Ethical Review Committee approved SAGE (RPC146), with local approval by the ethics review committee of the Chinese Center for Disease Control and Prevention (approval notice 200601). Written informed consent was obtained from each respondent.

### Outcome measures

In this study, The following tests are taken to measure cognitive performance: verbal recall(immediate and delayed), verbal fluency and digit span.

#### Verbal recall

The immediate and delayed verbal recall test were administered to assess learning capacity, memory storage and memory retrieval. In the test, the interviewer read a list of words, then the respondents were given one minute to repeat as many words as possible.This test was repeated three times. After about 10 minutes, the respondent was asked to again recall the words. Each word the respondent recalls correctly as well as any substituted words was recorded as one point.

#### Verbal fluency

The respondents were given one minute to tell the names of as many animals (including birds, insects and fish) that they can think of. One point was scored for each correctly named animal.

#### Digit span

Digit span test was administered to assess concentration, attention and immediate memory. The respondents were asked to repeat a string of numbers in a forward and backward order, respectively. The number of the longest series repeated without error were recorded as the total score.

#### Overall cognition score

An overall cognition score was created to represent respondents’ overall cognition status. The overall cognition score was based on the seven separate components of the cognition tests, including the four verbal recall trials, the verbal fluency test, the forward digit span test and the backward digit span test. Factor analysis was applied to evaluate and generate a single overall score. Higher scores reflected better cognitive performance.

#### Other covariates

Other variables used in the analyses included age, gender, marital status.

### Statistical method

A two-level hierarchical linear model was used to evaluate the association between SES at two levels (individual and community) and the overall cognition score. We chose the secondary sampling unit (SSU) level to represent the community level in our model, as no direct variable on community level SES was collected. We use weighted median years of education and median household income calculated in each SSU to represent the SES of these communities.

The equations at level-1, and 2 in our multivariate models are shown as below:

Level-1:

Yjk=α0j+α1Xjk+ejk (1)

Where Yjk is the outcome measure for subject k in community j (j = 1, … 127), Xjk is the individual level risk factors Xjk, and ejk is the error term following i.i.d. normal distribution N(0, e2).

Level-2:

α0j=β00+ β01Zj+εj (2)

Where Zj is the SSU community level risk factors, and εj is the random effects capturing the unexplained deviation of community j from the average level of all communities, which follows i.i.d. normal distribution N(0, ε2).

Covariates of interest at the individual level (level-1) include age, gender, marital status, years of education and household monthly income. we also included numbers of people in household to adjust for the effect of household size. Covariates of interest at the community level (level-2) include SSU-specific median years of education and median household income calculated. All models are stratified by residence (urban/rural). All analyses were performed using STATA v13.0.

## Result

The sample demographic characteristics are shown in Table 1. The proportion of women (53.1%) was higher than men (46.9%) in the study, with small sex differences by age groups and residence. The majority of the respondents were between 50 to 59 years old (43.3%); nearly half of all respondents (51.2%) lived in a rural area. The median education levels by residence are 7.5 years in urban and 2.7 years in rural areas. Fifty-seven percent had completed primary school or beyond. People who never received formal education accounted for 25.6% of respondents, while university graduates or higher accounted for 4.6%. The overall level of education for women was lower than that of men; over half of women in rural areas had no formal education, which was more than double the level for men in rural areas and women in urban areas. As household income quintile increased from low to high, the proportion of households steadily increased among urban but decreased in rural area.

**Table 1:** Demographic Characteristics at Individual, Household and Community Level.

	Characteristics	Urban		Rural	
		Women	Men	Women	Men
**Individual Level**	**Age group (n (%))**				
	50–59	1464 (40.9)	1062 (37.4)	1598 (46.8)	1571 (47.3)
	60–69	1036 (29.0)	837 (29.4)	1016 (29.8)	1030 (31.0)
	70–79	860 (24.0)	753 (26.5)	591 (17.3)	566 (17.0)
	80+	216 (6.0)	191 (6.7)	209 (6.2)	157 (4.7)
	**Education**				
	In years (mean (SD))	6.7 (4.6)	8.8 (4.2)	2.3(3.0)	4.6(3.4)
		n = 3557	n = 2827	n = 3393	n = 3292
	No formal education (n (%))	711 (19.9)	191 (6.7)	1740 (50.9)	707 (21.3)
	Less than primary (n (%))	406 (11.3)	209 (7.3)	835 (24.4)	898 (27.0)
	Primary school completed (n (%))	628 (17.5)	498 (17.5)	529 (15.4)	937 (28.2)
	Secondary school completed (n (%))	938 (26.2)	828 (29.1)	257 (7.5)	586 (17.6)
	High school completed (n (%))	704 (19.7)	723 (25.4)	60 (1.8)	193 (5.8)
	College completed or higher (n (%))	195 (5.4)	397 (14.0)	1 (0.03)	4 (0.1)
	**Number of people in Household** mean (SD)	2.69 (1.32)		2.63 (1.33)	
		n = 4247		n = 4338	
	**Income quintile** (n (%))				
	Lowest (<417)	215 (5.2)		1509 (37.7)	
	Second (417–999)	512 (12.3)		1035 (25.9)	
	Middle (999–1987)	936 (22.6)		801 (20.0)	
	Fourth (1987–3596)	1245 (30.0)		407 (10.2)	
	Highest (> 3596)	1240 (29.9)		251 (6.3)	
**Community Level**	**Median income** mean(SD)	2491.4 (1191.5)		903.7(667.0)	
		n = 63		n = 64	
	**Median education in year** mean(SD)	7.5 (2.1)		2.7 (1.4)	
		n = 63		n = 64	

The model coefficients for each of the variables in relation to overall cognitive score are shown in Table 2. An intercept only model is performed as Model 0. For urban, the Intraclass Correlation IC_community_ = 0.1207 indicating that 12.07% of the variance in overall cognition score could be explained by living in different communities. Model 1 added individual level demographic characteristics and household income. The results from multilevel model show that age, gender (only among rural participants) and marital status, as control variables, were all significantly related to overall cognition score. Model 2 added community level factors on the basis of Model 1. leading to an improvement in the BIC score. For all 2 models, at individual level, "years of education" was significantly associated with overall cognition score for both urban and rural areas (Model 1, 2). At the community level, a positive association was obtained between median household income, median years of education and overall cognition score in both urban and rural areas. (Model 2) however, for rural, the bigger BIC score of model 2 revealed that adding community level variables failed to improve the fit of the model.

**Table 2:** Multilevel analysis of Overall Cognition Score and related factors.

	Urban			Rural		
	Model 0	Model 1	Model 2	Model 0	Model 1	Model 2
	β(standard deviation)	β(standard deviation)	β(standard deviation)	β(standard deviation)	β(standard deviation)	β(standard deviation)
**Fixed Effect**						
Intercept	0.18(0.04)***	0.25(0.05)***	-0.02(0.05)	-0.17(0.03)***	-0.21(0.04)***	0.04(0.08)
age		-0.32(0.01)***	-0.32(0.01)***		-0.31(0.01)***	-0.31(0.01)***
Gender						
Female vs. Male		-0.002(0.02)	-0.002(0.02)		-0.03(0.02)	-0.02(0.02)
Marital status						
Never married/Separated/Widowed vs. Married/ Cohabitating		-0.08(0.03)**	-0.09(0.03)**		-0.18(0.03)***	-0.18(0.03)***
Years of education(centered)		0.20(0.01)***	0.20(0.01)***		0.27(0.01)***	0.27(0.01)***
Household size		-0.01(0.01)	-0.01(0.01)		0.004(0.01)	0.002(0.01)
Household income(Per 1000RMB)		0.002(0.01)	0.002(0.01)		-0.04(0.03)	-0.04(0.03)
Median household income (community, Per1000RMB)			0.10(0.05)*			0.21(0.07)**
Median years of education(community)			0.26(0.06)***			0.15(0.07)*
**Random Effects**						
Variance (community level)	0.1207(0.0232)	0.1353(0.0255)	0.0702(0.0139)	0.0655(0.0132)	0.077(0.0148)	0.0616(0.0121)
Variance (Residual)	0.8475(0.0156)	0.652(0.012)	0.652(0.012)	0.9088(0.016)	0.6955(0.0123)	0.6952(0.0123)
BIC	15,886.92	14,608.68	14,587.73	17,975.29	16,294.50	16,314.02
N	5,956	5,950	5,950	6,496	6,495	6,495

*P<0.05,

**P<0.01,

***P<0.001.

## Discussion

Significant associations between SES in both individual-level and community-level (only among urban participants) and cognitive performance were found in this study of a national sample of 13,157 Chinese aged 50 years and older, the largest China-based study to examine the cross-sectional relationship on this issue. The association was robust, remaining significant even after adjusting for demographic characteristics.

This study confirmed evidence from previous studies [9–14] that higher individual-level education is associated with higher cognitive performance. A comparable study among the Chinese population in Taiwan, also found low education was associated with a higher risk of cognitive impairment [15]. Two possible mechanisms may explain the relationship between educational attainment and cognitive function: first, those well educated people presumably have a larger brain reserve capacity than people with less schooling, which is the ‘brain reserve capacity’ hypothesis [16]. Well educated people have an advantage over the people with less schooling in searching for mental stimulation, which may lead to beneficial change of structure and function in the brain [17]. Alternately, education may mediate one's behavior to some extent, and could enhance general health, especially cognitive functioning[18].For example, people with higher education might adopt healthier lifestyles that are associated with good cognitive ability.

Although numerous studies have established a clear link between educational attainment and cognitive functioning at individual-level, only a few known studies are available in China that have investigated this association at community-level. A strong association between community level education and cognitive performance were found among urban participants even after adjusting for demographic characteristics in this study. (Although results of our study show that SES at community level is statistically significantly associated to cognitive performance for both urban and rural, this effects in rural area were not supported by BIC score, it’s likely that the significant effects were resulted from large sample size of our study.) Higher neighborhood socioeconomic deprivation was found to be related to lower cognitive functioning in one cross-sectional study [19], which is consistent with our findings. A study among community-dwelling older adults in Singapore also showed that neighborhood deprivation in urban areas is associated with cognitive function in older adults independent of the effects of individual and household socioeconomic factors[20]. One possible theory is that persons living in areas with generally low average education levels are possibly more likely to be exposed to the poor living conditions that may create hazards such as unemployment and low income, which thought to be always connected with physical and social resources scarcity, and furthermore affect engagement in both the physical and social activities that have been related to better cognitive functioning[21–23].

Our study revealed that financial condition, based on household income (particularly among urban participants) and community-level median household income, was associated with cognitive performance in urban area. Many previous studies in China and other countries have found that poor financial status was associated with worse cognitive function at the individual level [6, 24, 25]. In this study, this association was also found at community-level. A study focusing on socioeconomic status and cognitive function among community-dwelling older adults in Singapore showed that living in a low-SES community is independently associated with cognitive impairment[7]. This may be explained by the differences in distribution of public cultural facilities such as museums, bookstores, libraries, etc. which is also considered to have stimulating effect on the cognitive function maintenance and improvement [26, 27].

The strengths of our study are the sample size, the fact that the data were derived from a large, national probability sample of older adults and the use of a measure of cognition based on assessment of several cognitive domains. There are also limitations to acknowledge. First, the relationship between education attainment and cognition is much more complicated than we imagined, some experts suggested that education is causal [28], although other scholars believed education is an outcome of intelligence[29]. This study can only state association, but cannot infer causality, in part because of the cross-sectional data used in this study SAGE Wave1, but also because it would be difficult to obtain data about cognitive ability prior to participants' education, As such, we are unable to control for reverse causation because higher pre-existing cognitive ability might be the cause of both educational attainment and higher later-life cognitive ability.[30] the second and third waves of SAGE will may provide an opportunity to examine the direction and strengths of causal influences. Second, our results may be somewhat biased toward a cognitively well-functioning population, not only because it is a community-dwelling sample, but also because this analysis did not include the proxy-assisted interviews.

China’s population structure is changing rapidly, concurrent with expanding economic and social development inequality among different regions, which may be associated with cognitive functioning of older Chinese adults. Hence, understanding the role of socio-economic factors in determining health in older adults is essential in allocating resources appropriately. Identifying community-based SES variables that are associated with cognitive performance in the older population provides further evidence for the need to address community characteristics associated with deprivation. Future research should consider examining the longitudinal relationship between living in a lower SES neighborhood and risks for steeper cognitive declines, controlling for as many potential confounding factors at individual level as possible.

## Supporting Information

S1 Appendix Sampling and Weights. (PDF)

S1 Dataset Dataset. (DTA)

## Abbreviations

SAGE: Study on Global AGEing and Adult Health
SES: socio-economic status
PSU: principle sample units
SSU: secondary sample units
TSU: tertiary sample units
