# Supplementary material for: Social-Economic Status and Cognitive Performance among Chinese Aged 50 Years and Older
Source: PLoS One. 2016 Nov 18;11(11):e0166986. doi: 10.1371/journal.pone.0166986 (PMC5115845; doi:10.1371/journal.pone.0166986)
Supplement: S1 Appendix — (PDF) [file pone.0166986.s001.pdf]

Supporting information Appendix S1 **Sampling and Weights**

SAGE Wave 1

Sampling

The People's Republic of China administers 22 provinces, four municipalities, five autonomous regions and two special administrative regions. A province is an administrative division. Provinces were divided into three groups (central, eastern and western) based on their economic development and geographic location. Of the 22 provinces, 10 are classified as central, eight as eastern and four as western. Seven provinces were selected at random: two central, two western and three eastern provinces. Shanghai which is a municipality was selected with certainty.

China implemented a stratified, multistage cluster sampling design. The sample was stratified by provinces, eight in total, and is nationally representative. Guangdong, Shandong, Zhejiang and Shanghai are located in the east and represent the developed provinces of China. Hubei and Jilin are located in the middle region of China. Hubei, though it is located in eastern China, its economic status is similar to that of the middle provinces of China. Shaanxi and Yunnan provinces are located in western China and represent the less developed provinces.

One county in the rural regions and one district/city in the urban regions, was randomly selected from each province. Anji, Gucheng, Nanxiong, Meixian, Ji'an, Luwan, Ruschan and Tonghai were the urban cities selected while Wucheng, Wujiagang, Tianhe, Wangyi, Fengman, Qingpu, Lixia and Panglong the rural counties.

Four townships in each selected county and four community blocks in each selected city were selected probability proportional to size and constituted the PSUs. The measure of size being the number of households in the township or community block.

Two villages in each sampled township and two neighbourhood communities in each community block were selected probability proportional to size and constituted the SSUs. The measure of size being the number of households in the village or neighbourhood communities.

From each village and each neighbourhood community, two residential blocks were selected using cluster random sampling.

The CDC maintains an updated list of all households with persons aged 50 years and older (50+). Thirty-five households were randomly selected from each residential block using this list. The CDC also maintains an updated list of all households with persons aged 18-69. Excluding the households already selected seven households with a person aged 18-49 were randomly selected from the second list.

Eighty four households in total were randomly selected from each sampled village or neighbourhood community forming the TSUs. In the final stage, in an 18-49 household, one eligible member was selected using Kish tables. All

members aged 50 years and older were eligible from 50+ households (with multiple individual interviews possible).

Stages of selection

Strata: Province = 8

PSU: Township or Community = 64 surveyed

SSU: Village or Neighbourhood Community = 127 surveyed

TSU: Household = 10 752 surveyed

QSU: Individual = 15 050 surveyed

Weights

Household weights for analysis at household level and individual weights for analysis at person level were calculated. These were based on the selection probability at each stage of selection. Household weights are post-stratified by province and locality according to the 2008 household projections provided by China CDC and weight up to the total number of households in the eight provinces. Individual weights are post-stratified by province, sex and age groups (18-49, 50-59, 60-69, 70+) according to the 2008 population projections provided by China CDC and weight up to the total number of persons aged 18+. A second set of household and individual weights are available which are post stratified to weight up to the entire household and 18+ populations respectively in the PRC (nationally representative of these populations). Hong Kong and Macau are excluded from national totals. Weights are not normalized.
